# Traditional Chinese medicine on treating premenstrual syndrome and premenstrual dysphoric disorder: A protocol for systematic review and meta-analysis

**DOI:** 10.1097/MD.0000000000022694

**Published:** 2020-10-16

**Authors:** Mingzhou Gao, Hui Sun, Wenjun Sun, Dongmei Gao, Mingqi Qiao

**Affiliations:** a College of Traditional Chinese Medicine, Shandong University of Traditional Chinese Medicine, Jinan, Shandong Province; b School of Pharmaceutical Sciences, South-Central Minzu University, Wuhan, Hubei Province; c Research and Innovation team of Emotional Diseases and Syndromes in Shandong University of Traditional Chinese Medicine, Jinan, Shandong Province, China.

**Keywords:** premenstrual dysphoric disorder, premenstrual syndrome, systematic review, traditional chinese medicine

## Abstract

**Background:**

Premenstrual syndrome (PMS) and premenstrual dysphoric disorder (PMDD) are common disorders that manifest themselves in the late luteal phase, and significantly interfere with an individual's daily activities. Clinical evidence suggests that traditional Chinese medicine (TCM) may ease PMS/PMDD symptoms. Here, we review a protocol for exploring the effectiveness and safety of TCM in PMS/PMDD management.

**Methods:**

We will conduct a literature search for randomized controlled trials (RCT) for TCM use in PMS/PMDD on PubMed, web of science, EMBASE, the Cochrane central register of controlled trials (Cochrane Library), Chinese national knowledge infrastructure, Chinese VIP Information, Wanfang, as well as Chinese biomedical literature database. The search included all relevant reports for up to June 1, 2020. The search results were independently analyzed by 2 reviewers who extracted the data. RCT quality will be assessed using the risk-of-bias tool. The evidence will be inspected using the grading of recommendations assessment development and evaluation (GRADE). We will utilize Stata and Revman for systematic review and meta-analysis and analysis of direct and indirect evidence.

**Results:**

Based on current evidence, this study will elucidate the rationale for the utilization of TCM in PMS/PMDD treatment.

**Conclusion:**

Conclusions from this study will inform about the effectiveness and safety of TCM in PMS/PMDD management.

**Trial registration number:**

CRD42020192822.

**Ethics and dissemination:**

Since all data utilized in this systematic review and meta-analysis are published, ethical approval is not needed. Additionally, in the trial of the review process, all data will be evaluated anonymously.

## 1 Introduction

Premenstrual syndrome (PMS) and its clinically severe form, premenstrual dysphoric disorder (PMDD), are common psychological and somatic disorders with diverse clinical manifestations. PMS/PMDD symptoms include irritability, depression, mood swings, and sleep disturbances in women of reproductive age.^[1,2]^ Three percent to 8% of women in the reproductive age match the DSM-5 for PMDD, although its prevalence may be higher.^[3–5]^ Recent studies indicate increasing risk of suicidal behavior^[6]^ and worsening distance of menstrual symptoms in the general population, which affect 1 in 3 women.^[7]^

The underlying mechanisms of PMS/PMDD are unclear but genetic factors, negative cognitive styles, traumatic events, and preexisting anxiety disorders are thought to be PMDD risk factors.^[8–10]^ Mounting evidence suggests that brain abnormalities, including abnormal brain structure and function and inflammation, may contribute to PMS/PMDD etiology.^[11–13]^ Moreover, elevated cerebellar gray matter volume, as well as metabolism, has been detailed in PMDD. Additionally, differential corticolimbic stimulation in response to emotional stimuli differentiates the PMDD brain, particularly amplified amygdalar and reduced frontocortical role.^[14]^ Circulating ovarian steroids, particularly progesterone and its metabolite allopregnanolone, influence affective disorders.^[15]^ Bixo et al^[16]^ showed that allopregnanolone triggers negative mood symptoms in PMDD via its antagonizing effects on the GABAA receptor, suggesting altered sensitivity to allopregnanolone during PMDD.^[17]^

The symptoms of severe PMS/PMDD may vary. Fluoxetine is Food and Drug Administration-approved as first-line PMS/PMDD treatment.^[18]^ Multiple randomized controlled trial (RCTs) show that UC1010, vitex agnus castus, and adjunctive quetiapine SR are efficacious treatments.^[19,20]^ Since risk of impairment differs between symptom clusters, further research on individualized treatment is needed.^[21]^ TCM, including herbal medicine, has been utilized for the deterrence, treatment, as well as cure of multiple disorders and diseases for centuries.^[22]^ An epidemiological study by Mingqi Qiao proposed 2 PMDD subtypes based on TCM theory.^[23]^ Here, we purpose to inspect the efficacy, as well as the safety of TCM in PMS/PMDD management using network meta-analysis. Although systematic reviews on the utilization of herbal medicine for PMS treatment are available,^[24]^ the proposed study will have an expanded scope to determine the safety and efficacy of TCM in PMS/PMDD treatment.

## 2 Methods

### 2.1 Registration

This systematic review protocol is catalogued in PROSPERO (ID: CRD42020192822) and adheres to Cochrane handbook for systematic reviews of interventions, and preferred reporting items for systematic reviews and meta-analysis protocol (PRISMA-P) guidelines.

### 2.2 Inclusion criteria

#### 2.2.1 Types of studies

All RCTs involving PMS/PMDD treatment with TCM will be included. Included trials must clearly report randomized methods, TCM treatment information and indices, diagnostic maxims (ACOG for PMS, DSM-5 for PMDD), and efficacy evaluation. No restrictions are imposed on publication. Experiments are limited to human subjects. Language is restricted to Chinese and English. Non-RCT, quasi-RCT, case report series, and cross-study will be excluded.

#### 2.2.2 Subjects

Participants will have been definitely diagnosed with PMS/PMDD (refer to DSM-5). Studies involving psychiatric patients, subjects with severe physical illness, drug abuse history (constituting drugs for PMDD management within 3 months), hematological diseases, pregnancy, or suckling will be excluded. No restrictions are imposed on regions, citizenship, nationality, and case source.

#### 2.2.3 Types of Intervention

Various drug ingredients, TCM doses, or their combination with western medicine are employed as experimental interventions. Prescription drugs and proprietary Chinese medicines will be included. Other traditional Chinese therapies, including intravenous drugs, acupuncture, and massage, will be restricted. People on simple western medicine can be employed as control interventions. Alternatively, untreated subjects may be used as controls.

#### 2.2.4 Types of outcome measures

##### 2.2.4.1 Primary outcomes

Overall premenstrual symptoms will be inspected using a validated prospective screening tool like premenstrual symptom screening tool or daily record of severity of problems, or by predefined medical diagnostic criteria. It may be reported as an overall score or on separate subscales, such as mental and physical symptoms.

##### 2.2.4.2 Secondary outcomes

Secondary outcomes constitute specific PMS symptoms, including behavioral, psychological, and physical symptoms, treatment response rate depending on how it is examined in respective studies, and quality of life as measured using a validated scale like health-related quality of life).

### 2.3 Literature search

A literature search of articles about TCM use in PMS/PMDD published in the period up to June 2020 will be done on PubMed, Chinese national knowledge infrastructure, web of science, Cochrane central register of controlled trials (Cochrane Library), Chinese VIP information, Wanfang database, EMBASE, as well as Chinese biomedical literature database. The following search terms will be utilized: Premenstrual Syndromes OR Premenstrual Tension OR Premenstrual Tensions OR Premenstrual Dysphoric Disorder OR Premenstrual Dysphoric Syndrome ORPMS ORPMDD) AND (traditional Chinese medicine OR Korean medicine OR kampo medicine OR traditional medicine OR herbal medicine OR decoction OR proprietary Chinese medicine OR Chinese herbal medicine). Topics and abstracts regarding research on Chinese Journal of Male Science, Chinese TCM, and TCM, will be manually searched.

### 2.4 Data collection and evaluation

#### 2.4.1 Study selection

Endnote X 9.3.3 was used for preliminary evaluation of the titles, as well as abstracts based on the aforementioned inclusion maxims and eligible studies selected. Next, full texts of enrolled literature will be inspected and uncontrolled studies, nonrandomization studies, studies with inconsistent assessment maxims, and studies with similar data eliminated. Differences in opinion that emerge during this phase will be further discussed to reach a consensus. If differences cannot be resolved, a 3^rd^ investigator will intervene.

#### 2.4.2 Data mining and management

**Figure 1:**
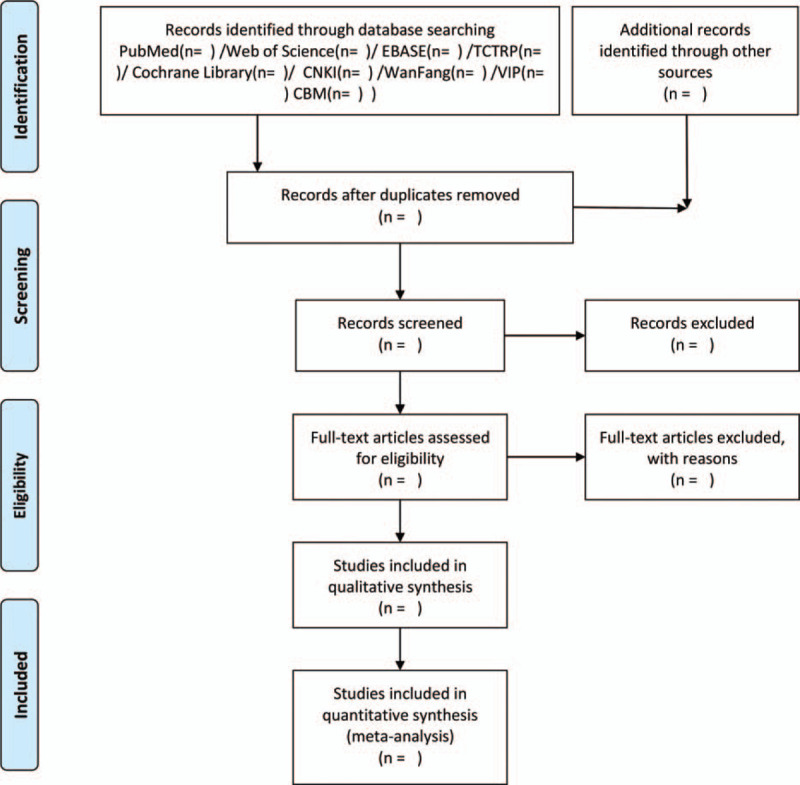
Flow chart of identified studies. Adapted from Moher D, Liberati A, Tetzlaff J, Altman DG, The PRISMA Group (2009). *P*referred *R*eporting *I*tems for *S*ystematic Reviews and *M*eta-*A*nalyses: The PRISMA Statement. PLoS Med 6(7): e1000097. doi:10.1371/journal.pmed1000097.

Data collection adhered to PRISMA guidelines. Using a data extraction table, 2 researchers independently mined information from enrolled literature. The composition of extraction included study design, randomized concealment and blinding, inclusion of basic case information, intervention strategies, observation indicators, and test results in treated versus control groups. For studies providing inception and postprocessing data, we will use the methods recommended by Cochrane to estimate changes. Information on the selection process is indicated on Figure 1.

#### 2.4.3 Risk of bias analysis

Two researchers will independently assess methodological integrity of the enrolled articles by employing the Cochrane's ROB tool, including if the random approach is correct, if blinding is employed, if it is hidden, if it is lost or withdrawn, if it is used for analysis, and whether results are accurate. Studies will be grouped into low-risk, high-risk, or unclear based on appropriate standards in the Cochrane intervention system assessment manual.

#### 2.4.4 Dealing with missing data

In case of lost or insufficient trial data, we will contact the study authors by email or phone for clarification. If sufficient data are unobtainable, the unusable data will be discarded.

#### 2.4.5 Statistical evaluations

Numerical variables will be indicated as a normalized mean difference with confidence intervals of 95%. Diversity in each pair comparison will be inspected using the *χ*^2^ assessment (test level a = 0.1). In the absence of diversity, a fixed-effect model will be utilized. In case of remarkable diversity between a group of studies, we will inspect the basis for the presence of heterogeneity by evaluating subjects’ characteristics and the level of intervention change. Sensitivity assessments or meta-regression and subgroup analyses were conducted to inspect possible origins of heterogeneity where necessary. Qualitative funnel chart and graphic symmetry analyses will be conducted to inspect the publication bias. Quantitative approaches such as the Begg, as well as the Egger test, will be employed to inspect the publication bias in applications.

#### 2.4.6 Heterogeneity evaluation

Random-effect model for meta-analysis will be used. In case of remarkable heterogeneity linking a group of articles, we will inspect the sources of heterogeneity from various attributes, including subjects’ features and level of intervention change. Sensitivity evaluations or subgroup evaluations will be conducted as needed to clarify heterogeneity.

#### 2.4.7 Evaluation of reporting biases

If >10 studies can be conducted, we will use Egger method to evaluate the impact of funnel asymmetry on publication bias and small research. Since the asymmetry of funnel chart does not inevitably show publication bias, we will differentiate between distinct causes of asymmetry, such as poor methodological quality from real data heterogeneity.

#### 2.4.8 Quality of evidence grading

Quality of the evidence underlying primary results will be inspected using GRADE. The inspections will constitute bias risk, heterogeneity, indirectness, imprecision, and publication bias. Evidence quality will be grouped as “very low,” “low,” “moderate,” or “high.”

## 3 Discussion

Lifestyle changes and aging populations have contributed to the rising rates of PMS/PMDD.^[25]^ PMS/PMDD frequently causes physical, psychological, and social strain in women of childbearing age. Symptoms of premenstrual syndrome include bloating, breast tenderness, and irritability. Symptoms and their severity may vary from person to person. For some, premenstrual syndrome is so severe that it interrupts daily routines, including work and school. For others, the impact is small.^[26–28]^

Chinese medicine has developed over thousands of years. Chinese medicine practitioners use various physical and mental exercises, including acupuncture or herbal therapy, to solve health problems.^[29]^ TCM considers PMS/PMDD to be mainly related to liver's failure to discharge and store blood. Some people also view PMS and PMDD as a manifestation of a disharmony between liver and spleen. TCM prescriptions are based on both the “disease” and TCM syndrome. Thus, it is not uncommon to prescribe the same formulation for different diseases if they fall in the same TCM syndrome.^[30]^

With improved understanding of PMS/PMDD, clinical reports on PMS/PMDD treatment using traditional Chinese medicine are rising. Chinese medicine achieves good results against PMS/PMDD irrespective of syndrome differentiation and treatment or special diseases. To the best of our knowledge, the efficacy, as well as the safety, of traditional Chinese medicine in the treatment of PMS/PMDD has almost no comparison. Considering this is a systematic review, the review will update the summary of the current evidence on effectiveness of traditional Chinese medicine in PMS/PMDD treatment. This review will be updated regularly to inform clinical decisions and policies.

## Acknowledgments

The authors thank the editor and anonymous reviewers for their contributions to improving the integrity of this auricle.

## Author contributions

**Data curation:** Mingzhou Gao, Hui Sun.

**Formal analysis:** Mingzhou Gao, Hui Sun, Wenjun Sun.

**Funding acquisition:** Dongmei Gao, Mingqi Qiao.

**Review & editing:** Mingzhou Gao, Dongmei Gao.

**Writing – original draft:** Mingzhou Gao, Hui Sun.
